# Apatinib inhibits VEGFR-2 and angiogenesis in an *in vivo* murine model of nasopharyngeal carcinoma

**DOI:** 10.18632/oncotarget.17264

**Published:** 2017-04-20

**Authors:** Qiu-Xia Peng, Yun-Wei Han, Yan-Ling Zhang, Jie Hu, Juan Fan, Shao-Zhi Fu, Shan Xu, Qiang Wan

**Affiliations:** ^1^ Department of Oncology, The Affiliated Hospital of Southwest Medical University, Luzhou 646000, China; ^2^ Shandong University, Shandong 250100, China; ^3^ Department of Nuclear Medicine, The Affiliated Hospital of Southwest Medical University, Luzhou 646000, China

**Keywords:** nasopharyngeal carcinoma, apatinib, VEGFR-2, cisplatin, combined therapy

## Abstract

Angiogenesis is initiated by the activation of the vascular epidermal growth factor receptor-2 (VEGFR-2) by the vascular epidermal growth factor (VEGF) ligand. Overexpression of VEGFR-2 increases the growth of nasopharyngeal carcinomas (NPC). Apatinib (YN968D1) is a highly-selective inhibitor of VEGFR-2, but its effects on NPC have not been hitherto investigated. In the present study, CNE-2 NPC cells were xenografted into 132 nude mice, which were treated with one of 6 drug regimens of apatinib administered alone or in combination with cisplatin (DDP). The impact of treatment regimens on the growth, microvascularization, apoptosis, and metabolic response of tumors, as well as mouse survival was determined by histopathology, immunohistochemistry (VEGFR-2 and CD31), terminal deoxynucleotidyl transferase dUTP nick-end labelling (TUNEL), micro 18F-FDG PET/CT imaging and survival curves. Administration of apatinib alone inhibited tumor growth, reduced microvascular density, and facilitated the apoptosis of tumors. Tumors treated simultaneously with apatinib and cisplatin exhibited significantly-increased inhibition of tumor growth, prolonged survival time, decreased expression of VEGFR-2, reduced microvascular density, and frequency of apoptosis over standalone and sequential administration therapy. Tumors treated simultaneously with apatinib and cisplatin had the lowest uptake of FDG. Taken together, the simultaneous administration of apatinib and cisplatin improves the therapeutic efficacy over standalone treatments, which also led to improved efficacy over sequential administration regimens. VEGFR-2 is an important predictive marker for efficacy of apatinib treatment of NPC.

## INTRODUCTION

Nasopharyngeal carcinoma (NPC) has a particularly high incidence in southern China, Southeast Asia, and North Africa [1]. Almost 20% of NPC patients with locally-advanced disease present with distant metastases at diagnosis, which is the main cause of treatment failure of NPC [2]. Accordingly, chemotherapy is an important treatment modality for patients with advanced nasopharyngeal carcinoma. However; the relatively-high efficacy of chemotherapies has not translated to an improvement in the survival rates of patients with advanced NPC, and the side effects of such treatments may in fact exacerbate the prognosis [3–4]. The demand for novel drugs, and targeted therapies in particular, is therefore high, with a view to improving the survival of, and reducing the side effects within, patients with advanced NPC undergoing chemotherapy. In recent years, targeted therapies that inhibit the microvascularization of NPC tumors have demonstrated therapeutic effects and tolerable adverse reactions; Cetuximab, for example, is an anti-angiogenesis therapeutic antibody that has been approved by FDA for treating advanced NPC [5]. An array of targeted therapies would be preferable, however.

Angiogenesis is crucial for cell development and wound healing, and is implicated in the pathology of cancers. Vascular endothelial growth factor (VEGF) signaling plays an important role in angiogenesis, and vascular endothelial growth factor receptors (VEGFRs) are tyrosine kinases that function as key regulators of this process. The VEGFR family proteins consist of VEGFR-1, VEGFR-2, and VEGFR-3 [6]. Among them, VEGFR-2 is the principal mediator of VEGF-induced angiogenic signaling. When stimulated by VEGF, VEGFR-2 is auto-phosphorylated at the carboxy terminusand kinase-insert region, leading to pro-angiogenic effects [7, 8]. The phosphorylation of specific sites creates binding sites for the SH2 domains of various signaling molecules and has subsequent effects on cell proliferation, migration, permeability, and survival of the vascular endothelium [7, 9]. VEGF and its receptor are highly expressed in many tumor types, including NPC [10–11]. Accordingly, inhibition of VEGFR-2 signaling is an attractive target for therapeutic strategies to treat NPC

Apatinib is a selective inhibitor of the VEGFR-2 tyrosine kinase, and a potent inhibitor of angiogenesis and tumor growth [7]. By binding to VEGFR-2, apatinib reduces the expression of VEGFR-2, which inhibits the effects of VEGF binding and subsequent VEGFR-2 autophosphorylation [12]. In addition, apatinib-mediated VEGFR-2 inhibition also appears to inhibit downstream phosphorylated extracellular signal-regulated kinases. Through this inhibition, apatinib has antiangiogenic and antitumor activity [12]. In a phase III clinical trial, apatinib proved to have therapeutic efficacy for the treatment of terminal, chemotherapy-refractory gastric cancers [13]. Its application is being extended for treating non-small-cell lung, breast, and hepatocellular cancers in phase II and III clinical trials across the People's Republic of China. Some case reports have also shown that apatinib has antitumor efficacy against soft tissue sarcoma [14–16]. It is worth noting that anti-angiogenic therapies may work synergistically with conventional chemotherapy treatments [17–19]. Tian *et al*. demonstrated that apatinib combined with conventional chemotherapy was more effective in restricting the growth of non-small cell lung and colon cancer xenografts in nude mice than standalone apatinib [7]. On the contrary, however, Lin *et al*. showed that standalone apatinib was less effective for treating H22 solid tumors in mice than apatinib combined with elemene [20].

Cisplatin is the conventional, non-specific chemotherapy for treating NPC. However; it is largely unknown if targeted, anti-angiogenesis therapies can augment its action, and indeed the best combination of anti-angiogenic and conventional chemotherapeutic drugs is also unknown. More so, the best therapeutic administration sequence for apatinib, be it simultaneous or sequential administration, has not been explored. The present study, therefore, aimed to explore the impact of standalone apatinib and apatinib and cisplastin combination therapy on the growth of NPC xenografted tumors *in vivo*, with a view to identifying the optimal treatment regimen that would inform clinical practice.

## RESULTS

### Antitumor efficacy of YN968D1 combined with DDP

**Figure 1:**
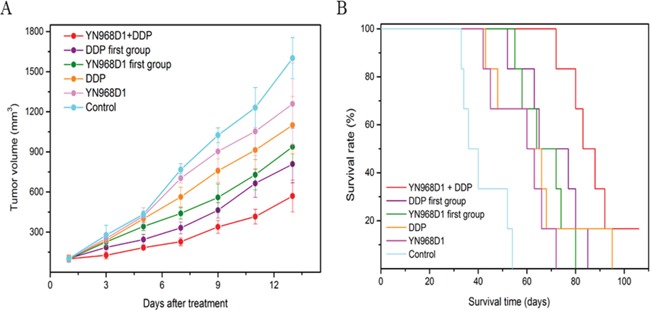
Tumor size and survival time of nude mice **(A)** Average volume of tumors xenografted into nude mice at 1 to 13 days post treatment **(B)** Kaplan-Meier survival curves associated with various treatment regimens.

The average volume of tumors xenografted into nude mice and treated with various drug regimens was determined (Figure 1A). Whilst apatinib modestly inhibited the growth of CNE-2 xenografted NPC tumors, treatment with a combination of apatinib and cisplatin significantly inhibited tumor growth, more so than that obtained by the two drugs administered alone(*P* < 0.05). Treatment with YN968D1 + DDP resulted in the most significant inhibition of tumor growth of all regimens, as determined by tumor volume (*P* < 0.05). Treatment first with YN968D1 or first with DDP, followed by either YN968D1 and DDP, did not have the same curative effect on mice as treatment with YN968D1 + DDP. Treatment administered as per the regimens of the YN968D1, DDP, YN968D1 first, DDP first, and YN968D1 + DDP groups inhibited tumor weights by 15, 31, 46, 53, and 70%, respectively. The Q index of the combinedYN968D1 + DDP treatment was 1.69, which indicated a synergistic effect when the drugs were administered simultaneously. However, the Q indexes of the DDP first (1.28) and YN968D1 first groups (1.13) alluded to an additive therapeutic effect when briefly administered simultaneously. There was no notable decrease in tumor weight resulting from standalone apatinib treatment. Whilst combined treatment with apatinib and cisplatin notably decreased the weight of tumors, the effect was short term and did not lead to an overall improvement in the survival time of nude mice.

The duration of survival of nude mice xenografted with CNE-2 NPC tumor cells and treated with different regimens was recorded, to which Kaplan-Meier survival curves were fitted (Figure 1B). The median survival time of control mice was 41 days. There was no significant difference in the median survival time of mice treated with YN968D1 (60 days) and DDP (67 days; *P* = 0.17), though these regimens significantly prolonged the median survival time over controls. Tumor-bearing mice treated with YN968D1 or DDP (first groups) had a longer median survival time over standalone treatments (69 and 71 days, respectively), though there was no significant difference between these regimens. Treatment with combined YN968D1 and DDP resulted in the greatest survival time (88 days) of all treatment regimens.

### Immunohistochemical and TUNEL analyses

**Figure 2:**
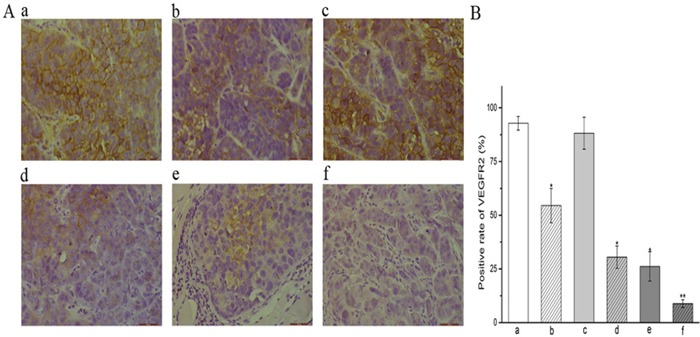
Expression of VEGFR-2 in CNE-2 NPC tumor tissue **(A)** Immunohistochemical staining of xenografted CN-2 NPCs treated with various treatment regimens with a VEGFR-2 antibody label (x400 magnification). **(B)** VEGFR2 positivity (%) within treatment groups.* *P* < 0.05 vs. control group. ** *P* < 0.05 vs. all groups. **(a)** control group; **(b)** YN968D1 group; **(c)** DDP group; **(d)** YN968D1 first group; **(e)** DDP first group; **(f)** YN968D1 + DDP group.

**Figure 3:**
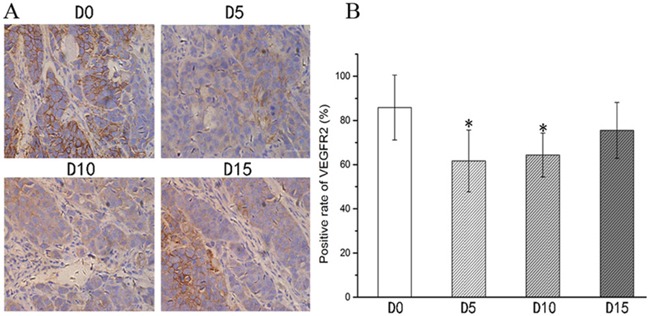
Expression of VEGFR-2 in CNE-2 NPC tumor tissue from D0, D5, D10 and D15 **(A)** Immunohistochemical staining of xenografted CNE-2 NPCs from D0, D5, D10 and D15 with an anti-VEGFR-2 antibody(x400 magnification). **(B)** VEGFR2 positivity (%) within D0, D5, D10 and D15 groups.* *P* < 0.05 vs. D0 group. * *P* >0.05 vs. D15 group.

Immunohistochemical staining of xenografted CNE-2 NPCs with a VEGFR-2 antibody revealed the impact of various treatment regimens (Figure 2). Whilst tumors of mice treated with the control or DDP were overwhelmingly positive, those treated with YN968D1 + DDP had only marginal staining. The percentage of VEGFR-2-positive cells in mice treated by apatinib(54.50%) was markedly less than the control (92.83%; *P* < 0.05) and cisplatin groups (88.16%; *P* < 0.05). There was no significant difference in the VEGFR-2 positivity of tumors treated with either control or cisplatin regimens (*P* > 0.05). Tumors treated with YN968D1 + DDP had the lowest proportion of VEGFR-2-positive cells of all treatment regimens (8.83%; *P* < 0.05). Immunohistochemical staining of xenografted CNE-2 NPCs from D0, D5, D10 and D15 with an anti-VEGFR-2 antibody revealed the activity of VEGFR-2 (Figure 3). The expression of VEGFR-2 in D0 and D15 groups was overwhelmingly positive, however, there was no significant difference in the percentage of VEGFR-2-positive cells between those two groups (85.83%,75.50%; *P* > 0.05). There was no significant difference in the VEGFR-2 positivity of tumors between the D5 (61.67%) and the D10(64.33%;P>0.05) samples. Furthermore, there was no significant difference in the percentage of VEGFR-2-positive cells in the D5, D10 and D15 groups (*P* > 0.05), but these were significantly lower than the D0 group (*P* < 0.05).

**Figure 4:**
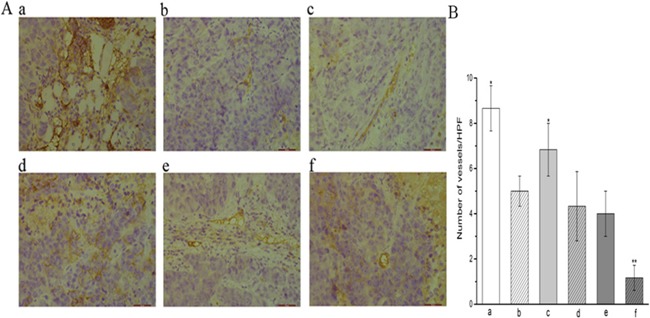
Expression of CD31 in CNE-2 NPC tumor tissue **(A)** Immunohistochemical staining of xenografted CN-2 NPCs treated with various treatment regimens with a CD31 antibody label (x400 magnification) reveals differences in the number of microvessels (magnification). **(B)** the histogram of the number of vessels in each group. * *P* < 0.05 vs. control group. ** *P* < 0.05 vs. all groups. **(a)** control group; **(b)** YN968D1 group; **(c)** DDP group; **(d)** YN968D1 first group; **(e)** DDP first group; **(f)** YN968D1 + DDP group.

Staining of tumor xenografts with a CD31 enabled the determination of microvessel density (MVD), which we used as a marker of angiogenesis (Figure 4). The MVD within tumors of control mice (8.67) was markedly greater than that of tumors treated with all other regimens (*P* < 0.01). Tumors treated with YN968D1 + DDP had the lowest MVD (1.17) of all regimens, especially compared with the control group and DDP group (*P* < 0.01). There was no significant difference in the MVD of xenografts treated with YN968D1 (5.00), YN968D1 first (4.33), and DDP first (4.00).

**Figure 5:**
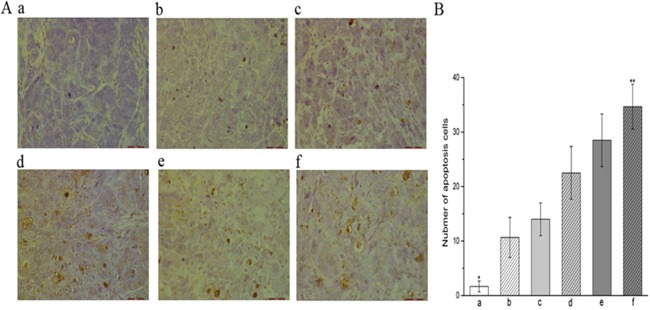
Tumor apoptosis detected by TUNEL assay **(A)** TUNEL of xenograft tumors treated with various regimens (x 400 magnification). Nuclei of apoptotic cells are stained brown, whereas unstained nuclei remain blue. **(B)** Frequency of apoptotic cells within xenograft tumors treated with various regimens. * *P* < 0.05 vs. all treatment regimens. ** *P* < 0.05 vs. all groups. **(a)** control group; **(b)** YN968D1 group; **(c)** DDP group; **(d)** YN968D1 first group; **(e)** DDP first group; **(f)** YN968D1 + DDP group.

Tumor xenografts treated with various regimens underwent TUNEL analysis for the determination of apoptotic cells (Figure 5). Positively-stained nuclei of apoptotic cells were sporadic within tumors of control mice and those treated with standalone regimens, whilst an increased frequency of positively-stained nuclei wasobserved in tumors treated with YN968D1 + DDP (34.67). The number of apoptotic cells was significantly lower in the control group (1.67) over all other groups (*P* < 0.05). Compared with the control and single treatment groups, combined treatment - particularly in the YN968D1 + DDP group - significantly increased the apoptosis of murine xenograft cells (*P* < 0.05).

### Micro 18F-FDG PET/CT imaging

**Figure 6:**
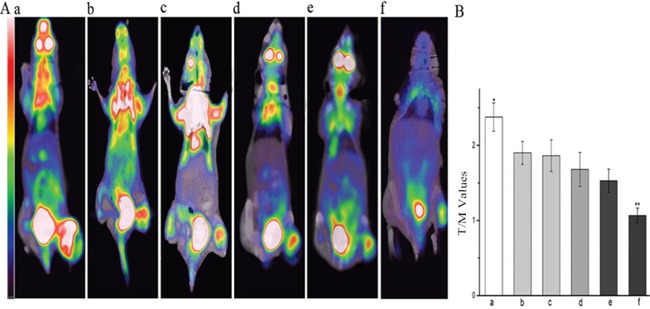
Micro 18F-FDG PET/CT imaging **(A)** Representative 18F-FDG PET scans of mice one day post treatment with various regimens. **(B)** T/M associated with various treatment regimens. * *P* < 0.05 vs. all the other treated group. ** *P* < 0.05 vs. all groups. T/M, tumor/muscle ratio; **(a)** control group; **(b)** YN968D1 group; **(c)** DDP group; **(d)** YN968D1 First group; **(e)** DDP First group; **(f)** YN968D1 + DDP group.

Xenografted mice underwent micro18F-FDG PET/CT imaging one-day post completion of treatment to discern the tumors early response to the various drug regimens (Figure 6). The metabolism of tumors treated with the control regimen was significantly higher than that of the other regimens (*P* < 0.05), whilst the metabolic signal of tumors treated with the YN968D1 + DDP regimen was notably lower (*P* < 0.05). The T/M associated with both treatment regimens was significantly lower than that of controls (2.35; *P* < 0.05). The T/M associated with tumors treated with YN968D1 + DDP was significantly lower than all other treatment regimens (1.06; *P* < 0.05). These results suggest that the NPC xenografts responded most favorably to treatment with YN968D1 + DDP.

## DISCUSSION

VEGFR-2 is typically overexpressed in NPCs, which in turn is a marker for poor outcomes [10, 21]. Apatinib is a highly-selective inhibitor of the VEGFR-2 tyrosine kinase, and thus we explored the impact apatinib administration on tumor growth in an *in vivo* murine model of NPC. We also sought to determine if apatinib, when administered simultaneously, would augment the therapeutic effect of cisplatin, and aimed to determine the optimum administration regimen. The present preclinical study is the first to investigate the efficacy of apatinib for treating NPC

The present study demonstrated that standalone apatinib therapy significantly inhibited the growth, in terms of tumor volume and weight, of NPC xenografts with tolerable adverse reactions relative to untreated controls. Furthermore, mice treated with apatinib survived for a significantly-longer period than untreated mice. 18F-FDG PET/CT imaging is widely used in clinical practice for monitoring the shift in glucose metabolism inherent in malignant cells - the so-called Warburg effect. Tumors with poor FDG uptake tend to respond more favorably to treatment, whereas tumors that rapidly uptake FDG, through an increase in glucose metabolism, tend to respond poorly to treatment and have a worse prognosis. The present study has shown that tumors treated with apatinib had a lower 18F-FDG uptake compared to untreated control tumors. Accordingly, we explored if the expression of VEGFR-2, density of microvessels, and presence of apoptosis could explain this phenomenon, and in particular if apatinib could inhibit angiogenesis. Staining with a labelled anti-VEGFR-2 antibody showed that VEGFR-2 was highly expressed within untreated CNE-2 nasopharyngeal carcinoma xenografts. Mice administered with apatinib had a significantly-lower expression of VEGFR-2 and microvascular density, and a markedly-increased frequency of apoptotic cells compared to untreated controls. This finding confirmed that apatinib has a clear inhibitory effect on VEGFR-2, which then reduced the density of microvascularization, thereby inducing apoptosis and inhibiting tumor growth. Based on measuring the activity of VEGFR-2, we found that after a few days of treatment with apatinib, VEGFR-2 expression was increased, suggesting that patients should take oral apatinib over a longer term to achieve long-term control of tumor growth. Our immunohistochemical and TUNNEL analyses therefore confirm the inhibitory effect of apatinib on NPC tumor growth. Furthermore, we show that VEGFR-2 is a predictive marker for the use of apatinib for NPC treatment, by discriminating patients who are most likely to benefit from apatinib from those that would be exposed unnecessarily to its toxicity [22].

Tian, *et al* showed that that apatinib combined with conventional chemotherapy was effective for the treatment of various malignant tumors xenografted in nude mice [7]. The present study explores the different administration sequences of apatinib and cisplatin on nasopharyngeal carcinoma xenografts in nude mice, with a view to determining the optimal drug combination and administration regime. Accordingly, subsets of 18 mice were treated with apatinib (first), cisplatin (first) or concurrent apatinib and cisplatin. Simultaneous treatment with apatinib and cisplatin had a synergistic effect, as determined from the volume and weight of treated tumors. However; the sequential administration of apatinib and cisplatin only demonstrated an additive effect. To further investigate the synergistic effect of apatinib combined with cisplatin on xenografted human nasopharyngeal carcinoma, micro18F-FDG PET/CT scans were obtained of treated mice, thereby enabling the assessment of the early tumor response. Median survival time was also noted. We found the lowest uptake of FDG, and the longest survival time of host mice, occurred in instances of simultaneous administration of apatinib and cisplatin, therefore alluding to its superiority over other treatment regimens. Our data also suggests that the simultaneous administration of apatinib and cisplatin can further enhance the therapeutic effect of apatinib over successive administration.

Our observations have shown that tumors treated with a combination of apatinib and cisplatin had the lowest expression of VEGFR-2 lowest density of microvascularization, and the highest frequency of apoptosis, compared with all other tested drug regimens. There are no reports of the mechanism of combinations of apatinib with other chemotherapy drugs leading to a synergistic effect. Through the above experiments, we propose the following hypothesis:1. apatinib inhibits the expression of VEGFR-2, thereby blocking angiogenesis. 2. cisplatin is inherently cytotoxic, inducing apoptosis;apoptosis of tumor cells leads to a simultaneous decrease in VEGF secretion and a decrease in VEGFR-2 expression, which leads to afurther inhibition of angiogenesis and tumor growth. These may be the reasons that apatinib combined with cisplatin produced a synergistic effect.

## MATERIALS AND METHODS

### Murine tumor models

Four-week old female nude mice (16.2 ± 0.82 g in weight) were purchased from Tengxin Biotechnology Co. Ltd. (Chongqing, China). Mice were housed at the animal research facility at The Affiliated Hospital of Southwest Medical University (Luzhou, China) in groups of five per cage, and were maintained under pathogen-free conditions. Mice were acclimatized to standardized laboratory conditions for at least a week prior to experimentation (24 ± 2°C; 50 ± 10% relative humidity; 12 h light–dark cycles). Mice were provided with standard laboratory food and tap water *ad libitum*.

Nude mice were inoculated with the CNE-2 NPC cell line by subcutaneous injection of 1 × 10^7^ CNE-2 cells (suspended in 0.1 mL 0.9% NaCl solution) at the dorsal aspect of the right foot. Tumor growth was evaluated every other day by measurement of the tumor diameter (mm) along the major (a) and minor axes (b), from which the tumor volume (V; mm^3^) was calculated according to the formula: V = 0.5 × a × b^2^. All animal procedures were approved by the Institutional Animal Care and Treatment Committee of Southwest Medical College.

### Experimental design

**Figure 7:**
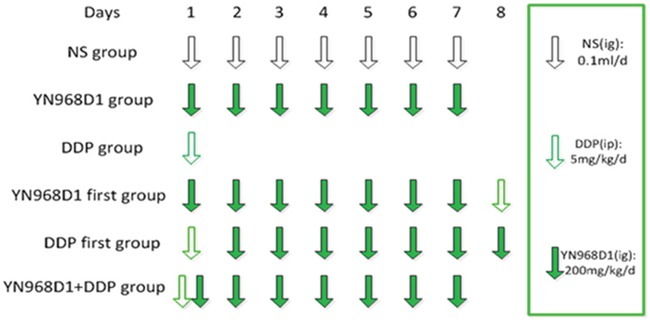
Treatment schedule Ten days after the average tumor volume reached 100 mm^3^, mice were randomized and received one of six treatment regimens over 8 consecutive days. I.p, intraperitoneal; I.g, intragastric.

Ten days after the average tumor volume reached 100 mm^3^, one-hundred and eight nude mice with tumor volumes of 100 mm^3^ were divided into 6 groups (n = 18 per group except for the YN968D1 group, which had forty-two nude mice), and were treated for 8 consecutive days as shown in Figure 7. Briefly, i) the control group was administered with 0.1mL normal saline per day on days 1 to 7; ii) the YN968D1 group received 200 mg/kg/day on days 1 to 7); iii) the cisplatin group received 5 mg/kg/day on day 1); iv) the YN968D1 first group received YN968D1 200 mg/kg/day administered on days 1 to 7 and cisplatin 5 mg/kg/day on day 8; v) the DDP first group received cisplatin 5 mg/kg/day administered on day 1 and YN968D1 200 mg/kg/day on days 2 to 8; and vi) the YN968D1 + cisplatin group was administered with YN968D1 200 mg/kg/day on days 1 to 7 and cisplatin 5 mg/kg/day on day 1. Apatinib and normal saline were received via intragastric administration, whilst cisplatin was administrated via intraperitoneal injection.

The day after the treatments ended, 6 mice from each group were sacrificed by cervical dislocation. In order to measure the activity of VEGFR-2, some nude mice were sacrificed from the YN968D1 group, 6 nude mice were sacrificed before the treatment(D0), 6 nude mice were sacrificed on the 5^th^ day (D5) of treatment, 6 nude mice were sacrificed on the 10^th^ day(D10) and the 15^th^ day (D15) after treatment. The tumors were then excised and weighed. A section of each tumor was fixed with 10% neutral formaldehyde in preparation for immunohistological analysis. The rate of tumor growth inhibition was calculated as follows: (1- weight of experimental group/weight of control groups) × 100%. The co-operation index (Q) was calculated as follows [24]: Q = E(A + B)/[EA + (1- EA) x EB], where E(A+ B) represents the inhibition rate of the drugs combined, and EA and EB represent the inhibition rate of the apatinib or cisplatin alone, respectively. A co-operation index (Q) 0.85 to 1.15 indicated an additive effect of the two drugs, whereas Q > 1.5 was indicative of a synergetic effect between the two drugs. The other 6 mice per group were observed for tumor growth and animal survival time. Tumor volumes and animal body weights were measured every two days using the previously described formula, V = 0.5 × a × b^2^, where *V* is the tumor volume, *a* is the length of the major axis and *b* is the length of the minor axis. Animals were observed until they died a natural death, and the average survival time of each group of nude mice was calculated.

### Immunohistochemical and TUNEL analyses

CNE-2 xenografts treated with 6 variable regimens were stained by immunohistochemical and terminal deoxynucleotidyl transferase biotin-dUTP nick-end labelling (TUNEL). All samples were stained with hematoxylin and eosin beforehand, and were histologically confirmed to be of NPC origin. Sections were labeled with either a VEGFR-2 (Bio-World, Dublin, OH, USA) or a rabbit anti-mouse anti-cluster of differentiation 31 (CD31) antibody (1:10 dilution; Bio-World, Dublin, OH, USA) for determination of VEGFR-2 expression and microvessel density, respectively, or underwent TUNEL labelling(Boehringer-Mannheim, Germany) for the detection of apoptosis. The expression of VEGFR-2 and CD-31 in tumors was examined by streptavidin-peroxidase (SP) immunohistochemical staining. All staining steps were carried out according to the manufacturer's instructions. Images were visualized under an optical microscope (Olympus, Tokyo, Japan).

Two skilled pathologists independently reviewed the slides and recorded the proportion of stained cells in each treatment group. For measurement of apoptosis or expression of VEGFR-2, five random fields from 6 slices were counted per group. TUNEL-positive brown nuclei within tissues were counted within a single high-power, x400 magnification field. The percentage (%) of VEGFR-2-positively stained, pale brown/brown cell membranes was determined within a single high-power, x400 magnification field. Microvessel density (MVD) was quantitated as per the method described by Weidner *et al*. [23]. Briefly, the tumor region that exhibited the most dense vascularization was identified at low magnification (x40), within which the number of positively-stained microvessels were counted within a single high-power, x400 magnification field. The MVD was expressed as the number of microvessels counted per field (n). CD31-stained endothelial cells, or endothelial cell clusters that were clearly separated from adjacent microvessels, tumor cells or connective tissue, were considered to be a single microvessel.

### Micro 18F-FDG PET/CT imaging

The early tumor response to treatment was determined by micro18F-FDG PET/CT images obtained with an Inveon micro PET/CT animal scanner (Siemens, Germany). Post treatment, 6 mice were fasted and anesthetized with 1% pentobarbital (5 mL/kg). Mice were then administered with an intravenous injection of 100–200 uCi 18F-FDG and positioned in the center PET field of view ring. PET/CT images (80 kV; 500 uA; 1.5 mm slice thickness; 10 min per bed position) were acquired 30 min post 18F-FDG administration. Data was acquired from image plane with the largest tumor appearance. An irregular region of interest (ROI) covering the entire tumor was drawn manually. In addition, ROIs were drawn on the contralateral paraspinal muscles. Uptake of 18F-FDG tracer in tumor and muscle tissues was determined in the attenuation-corrected, transaxial tomographic slices by calculating the standard uptake value (SUV) in a given ROI. The tumor/muscle (T/M) ratio was calculated from the maximal SUVs for 18F-FDG obtained from the selected ROI and contralateral paraspinal muscles, respectively.

### Statistical analyses

Data analyses were performed within SPSS software version 17.0 (SPSS, Inc., Chicago, IL, USA). All data are expressed as the mean ± standard deviation. Comparisons between multiple groups were made with the one-way ANOVA test. Survival curves were fitted based on the Kaplan–Meier method. *P* < 0.05 was considered statistically significant.

## CONCLUSIONS

In conclusion, the present study demonstrates that apatinib inhibits the growth of NPC xenografts in association with the downregulation of VEGFR-2, decreased angiogenesis and induced apoptosis. Furthermore, based on tumor volume and weight measurements, median survival time, the results of immunohistochemical staiing and apoptosis detection, as well as PET/CT imaging on the CNE-2 xenograft mode, simultaneous, rather than sequential administration of apatinib and cisplatin have greater efficacy for treating NPC. VEGFR-2 is an important predictive marker for the efficacy of apatinib in the treatment of NPC.
